# Association of scrub typhus with incidence of dementia: a nationwide population-based cohort study in Korea

**DOI:** 10.1186/s12879-023-08107-0

**Published:** 2023-03-01

**Authors:** Jooyun Kim, Hyeri Seok, Ji Hoon Jeon, Won Suk Choi, Gi Hyeon Seo, Dae Won Park

**Affiliations:** 1 Division of Infectious Diseases, Department of Medicine, Korea University Ansan Hospital, Korea University College of Medicine, 123 Jeukgeum-ro, Danwon-gu, Ansan, 15355 Republic of Korea; 2 Health Insurance Review and Assessment Service, 60 Hyeoksin-ro, Wonju-si, Gangwon-do 26465 Republic of Korea

**Keywords:** Scrub typhus, Tsutsugamushi, Dementia, Alzheimer

## Abstract

**Background:**

Scrub typhus is a mite-borne infectious rickettsial disease that can occur in rural and urban areas, with an especially high prevalence in older populations. This disease causes systemic vasculitis that can invade the central nervous system. Considering these characteristics, here we examined whether scrub typhus was associated with the occurrence of dementia, using large population-based cohort data.

**Method:**

This population-based cohort study enrolled patients aged 60–89 years using data from the Health Insurance Review and Assessment database of South Korea between 2009 and 2018. We defined scrub typhus and dementia using International Classification of Diseases, Tenth Edition diagnostic codes. The control group was stratified according to age and sex at a ratio of 1:5 to the case group in the study population. The index date was set after 90 days beyond the date of the scrub typhus diagnosis, while the observation period was from the time of the index appointment to December 31, 2020. The primary outcome was newly diagnosed dementia. The secondary outcome was dementia classification, such as Alzheimer’s disease, vascular dementia, and other. All analyses were conducted by matching age, gender, and comorbidity.

**Results:**

During the observation period, 10,460 of 71,047 (14.7%) people who had a history of scrub typhus versus 42,965 of 355,235 (12.1%) people in the control group, that is, with no history of scrub typhus, were diagnosed with dementia (adjusted hazard ratio, 1.12; 95% confidence interval, 1.10–1.15, p < 0.001). The Kaplan–Meier curves for time to cumulative incidence of dementia showed that the dementia incidence in both groups increased over time, while individuals with a past history of scrub typhus had a higher incidence of dementia than the control group. Second, the risk of Alzheimer’s disease was significantly higher among patients with a history of scrub typhus (adjusted hazard ratio, 1.15; 95% confidence interval 1.13–1.18, p < 0.001).

**Conclusion:**

In conclusion, a history of scrub typhus infection in old age is significantly associated with an increase in dementia, especially Alzheimer’s disease. Our results suggest that prevention and appropriate treatment of scrub typhus should be emphasized as a dementia prevention measure.

## Background

Scrub typhus is an infectious disease that is transmitted by human bites from mites infected with *Orienta tsutsugamushi*, a gram-negative coccobacillus in the family Rickettsiaceae. This bacterium infects host vascular endothelial cells and is released from infected cells, causing systemic vasculitis such as Scrub typhus [1].

Most cases are found in Southeast Asia, China, Japan, India, Indonesia and northern Australia, especially in rural areas and both residents and visitors are susceptible to infection [2]. In 2016, there were nearly 11,000 new cases in South Korea. Since then, more than 4,000 new cases have been reported annually [3]. In terms of age distribution, the proportion of affected older individuals aged 60 years and older is high [4, 5]. Unlike other infectious diseases, scrub typhus is characterized by systemic vasculitis. In the early stages of the infection, proper antibiotic treatment results in good responses. However, delayed treatment can lead to severe systemic progression and complications ranging from pneumonia and acute renal injury to invasion of the central nervous system (CNS) resulting in meningitis or encephalitis [6]. The role of scrub typhus in the long-term development of CNS complications remains unknown.

Dementia is a degenerative disorder that affects the brain, with more than 90% of cases diagnosed after 65 years old as the age is the leading risk factor. As a result, the increase in the mean population age worldwide is associated with a steady increase in the incidence and prevalence of dementia [7]. In 2015, according to the World Alzheimer Report, there are nearly 46.8 million dementia cases worldwide, which is expected to become 131.5 million by 2050 [8]. Although the pathogenesis of dementia remains unclear, it may be associated with comorbidities affecting the blood vessels, such as hypertension and diabetes [9, 10]. Other infectious diseases such as *Borrelia burgdorferi, Helicobacter pylori*, *Chlamydia pneumoniae* and herpes simplex virus type 1 (HSV-1) may also be associated with dementia [11–16].

Considering the characteristics of scrub typhus infection (occurring frequently in old age and involving the inflammation of systemic blood vessels that can invade the CNS), scrub typhus may be associated with the occurrence of dementia.

This study aimed to investigate this hypothesis using large population-based cohort data from the Health Insurance Review and Assessment Service (HIRA) in South Korea.

## Methods

### Data source

HIRA is the data source responsible for claim reviews and quality assessments of the National Health Insurance (NHI) and National Medical Aid (NMA) programs, of which all medical institutions and local pharmacies are compulsory participants. All medical expenditures within the NHI and NMA programs are monitored by HIRA for redemption purposes. The HIRA claims database contains basic information on patients’ sociodemographic characteristics and prescriptions, diagnosis and diagnostic procedures [17].

This dataset contains coded information for all of the diagnoses, tests, and treatments prescribed in South Korea since January 2007.

### Study population

We set the washout period for all factors that may affect the occurrence of dementia from January 2007 to December 2008.

From January 2009 to December 2018, among people aged 60 to 89, those diagnosed with scrub typhus were classified into the scrub typhus group. The index date was set after 90 days of scrub typhus diagnosis date to exclude the screening effect on dementia diagnosis. The operational definition of scrub typhus was as follows: (1) International Classification of Diseases, Tenth Edition (ICD-10) diagnostic code corresponding to scrub typhus (A75.3) during the registration period; and (2) prescription of doxycycline, the treatment of scrub typhus, for at least three days a week before or after the day when the diagnostic code was added.

The operational definition of dementia was as follows: (1) code for dementia (V-code V800, V801, V810, V811) in expending benefit coverage, reduces medical costs for patients with fatal diseases, during the follow-up period; or (2) at least one diagnostic code for dementia (F00.x, F01.x, F02.x, F03.x, G30.x), examination code (Mini-Mental State Examination [MMSE F6216] and one Global Deterioration Scale score [GDS, F6221] and Clinical Dementia Rating score [CDR, F6222]), or drug code (donepezil [1486, 6434], rivastigmine [2245], and/or galantamine [3852]) within the adjacent 4 weeks. In South Korea’s Health Insurance System, hospitals can receive medical expenses after the evaluation and approval of the Review and Assessment Service; it was considered that the possibility of falsely coding as dementia would be low.

We also classified patients by dementia subgroup into Alzheimer’s disease (AD), vascular dementia, and other, setting the operational definition of the subtype of dementia by using diagnostic codes. Among people diagnosed with dementia, those diagnosed with F00.x or G30.x code corresponding to Alzheimer’s were set as Alzheimer’s group, and those who received F01.x code corresponding to vascular dementia were set as vascular dementia group.

As a control group, among the data sets of HIRA, those aged 60–89 who were not infected with scrub typhus from January 2007 to December 2020 and applied to the exclusion criteria were randomly selected, stratified by age and sex at a ratio of 1:5.

We excluded those who were diagnosed with scrub typhus or dementia before the end of the washout period, for whom covariate data were missing, or who died during the observation period.

Institutional Review Board of Korea University Ansan Hospital (2022AS0001) and the HIRA (2022-001) approved the study. They also waived the informed consent to the study. We report the results in compliance with the Strengthening the Reporting of Observational Studies in Epidemiology (STROBE) reporting guidelines [18].

### Outcomes

The observation period was set from the index date to December 31, 2020. The primary outcome of this study was the event and timing of newly diagnosed dementia during the observation period. The secondary outcome was set as a subgroup of dementia that occurred during the observation period.

### Covariates

All variables that were used to treat the disease at least once between January 2007 and the index date were included as covariates. Age and sex were set at the time of the index date, and age was classified as 60–69 years or 70–89 years. The other covariates were defined as those with an ICD-10 diagnostic code. The analysis was adjusted for several known risk factors, such as age, sex, central degenerative disease (G20.x and G31.x), stroke (I63.x, I64.x, and I69.x), and hypertension (I10.x–I15.x), diabetes mellitus (E11.x-E14. x), and major depressive disorder (F32.x, F33.x) [14].

### Statistical analysis

We represent the Categorical variables using numbers (%) and mean ± SD for continuous variables. Fisher’s exact test, Student’s t-test and the chi-squared test were conducted for intergroup comparisons. Cox proportional hazards analysis for the incidence of dementia was performed to estimate the hazards of dementia incidence with the adjustment for age, sex, and comorbidities, and hazard ratios (HRs) were calculated with 95% confidence intervals (CI). The cumulative incidence curves of dementia were constructed and compared between groups using Kaplan–Meier curves. The follow-up period started on the index date and was censored on the date of diagnosis of dementia or the end of the study. All analyses were conducted by matching age, gender, and comorbidity. Statistical significance was considered when the two-tailed P value was < 0.05. On the other hand, R version 4.0.2 was the implemented statistical problem.

## Results

**Fig. 1:**
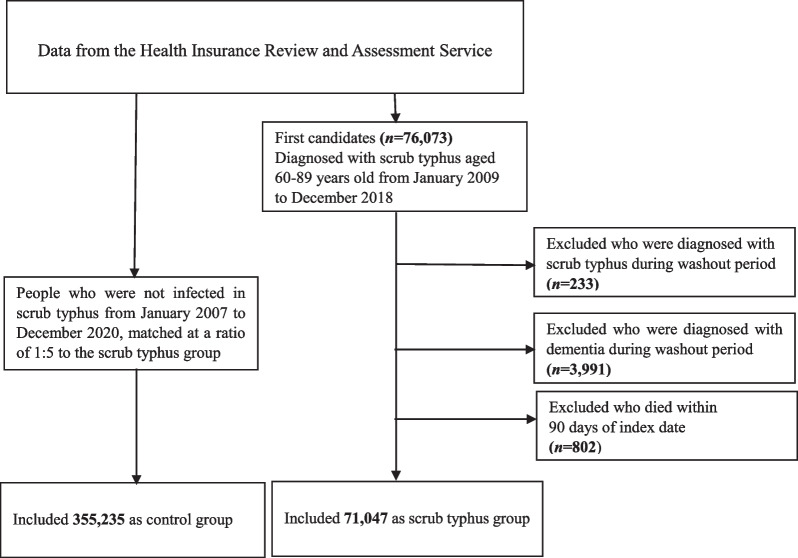
Flow chart of the study population

At the baseline, 76,073 individuals aged 60–89 years old were confirmed to have scrub typhus between January 2009 and December 2018. After the exclusion of participants who were diagnosed with scrub typhus during the washout period (n = 233), those diagnosed with dementia before the index date (n = 3991), and those who died before the index date (n = 802), a total of 71,047 subjects were included in the scrub typhus group. A control group was randomly selected at a ratio of 1:5 with sex and age matching; thus, 355,235 subjects were included (Fig. 1). The mean follow-up period was 5.6 ± 2.9 years. The case group was followed for 5.7 ± 2.8 years versus the control group for 5.6 ± 2.9 years.

All subjects of characteristics are presented in Table 1. Since the control group was selected by matching sex and age with the case group, the ratio of males to females was 37.3–62.7%. Likewise, for age, the ratios were the same between groups: 47.1% were 60–69 years old, while 52.9% were 70–89 years old.

**Table 1:** Participants’ characteristics values are shown as n (%) or mean ± standard deviation

	Total (*N* = 426,282)	Past history of scrub typhus (*n* = 71,047)	Non-history of scrub typhus (*n* = 355,235)	P value
Age				
Mean age (years ± SD)	70.6 ± 7.0	70.6 ± 7.0	70.6 ± 7.0	> 0.999
60–69 (*n*, %)	200,580 (47.1)	33,430 (47.1)	167,150 (47.1)	> 0.999
70–89 (*n*, %)	225,702 (52.9)	37,617 (52.9)	188,085 (52.9)	
Sex				
Male (*n*, %)	159,126 (37.3)	26,521 (37.3)	132,605 (37.3)	> 0.999
Female (*n*, %)	267,156 (62.7)	44,526 (62.7)	222,630 (62.7)	
Comorbidity (*n*, %)				
Stroke	78,923 (18.5)	13,804 (19.4)	65,119 (18.3)	< 0.001
Central degenerative disease	33,325 (7.8)	6876 (9.7)	26,449 (7.4)	< 0.001
Diabetes mellitus	219,506 (51.5)	40,010 (56.3)	179,496 (50.5)	< 0.001
Hypertension	286,724 (67.3)	47,067 (66.2)	239,657 (67.5)	< 0.001
Major depressive disorder	108,146 (25.4)	20,291 (28.6)	87,855 (24.7)	< 0.001
Dementia subtype				
All (*n*, %)	53,425 (12.5)	10,460 (14.7)	42,965 (12.1)	< 0.001
Alzheimer (*n*, %)	38,828 (9.1)	7770 (10.9)	31,058 (8.7)	
Vascular (*n*, %)	947 (0.2)	163 (0.2)	784 (0.2)	
Others (*n*, %)	13,650 (3.2%)	2527 (3.6%)	11,123 (3.1%)	
Follow-up duration (years ± SD)	5.6 ± 2.9	5.7 ± 2.8	5.6 ± 2.9	< 0.001

Regarding comorbidities, stroke (19.4% vs. 18.3%, p < 0.001), central degenerative disease (9.7% vs. 7.4%, p < 0.001), diabetes mellitus (56.3% vs. 50.5%, p < 0.001), and major depressive disorder (28.6% vs. 24.7%, p < 0.001) were significantly more prevalent in the case group. However, hypertension was more common in the control group (66.2% vs. 67.5%, p < 0.001).

Of the 426,282 participants, 53,425 (12.5%) were diagnosed with dementia during the observation period between the index date and December 31, 2020. In the scrub typhus group, 10,460 (14.7%) people were diagnosed with dementia, a significantly higher incidence comparing to the control group (n = 42,965 [12.1%]; p < 0.001) (Table 1).

Cox proportional hazards analysis revealed that dementia incidence was higher among individuals with a past history of scrub typhus than in the controls. Crude HR was 1.19 (95% CI 1.16–1.21, p < 0.001) and the adjusted HR (aHR) was 1.12 (95% CI 1.10–1.15, p < 0.001) (Table 2). When classified into subgroups, the risk of AD was higher in the scrub typhus group (aHR, 1.15; 95% CI 1.13–1.18, p < 0.001). However, there was no statistically significant differences regarding the vascular dementia risk (aHR, 0.98; 95% CI 0.83–1.17, p = 0.853) and other types of dementia (aHR, 1.05; 95% CI 1.00–1.09, p = 0.033).

**Table 2:** Multivariate Cox proportional hazards analysis of dementia by subtype

	patients (*n*, %)		Crude HR	P value	Adjusted HR*	P value
	Scrub typhus group	Control group				
Dementia subtype						
All	10,460 (14.7)	42,965 (12.1)	1.19 (1.16–1.21)	< 0.001	1.12 (1.10–1.15)	< 0.001
Alzheimer’s disease	7770 (10.9)	31,058 (8.7)	1.22 (1.19–1.25)	< 0.001	1.15 (1.13–1.18)	< 0.001
Vascular	163 (0.2)	784 (0.2)	1.02 (0.86–1.20)	0.837	0.98 (0.83–1.17)	0.853
Others	2527 (3.6)	11,123 (3.1)	1.11 (1.06–1.16)	< 0.001	1.05 (1.00-1.09)	0.033

*Model adjusted for age, sex, and comorbidities (Stroke, Central degenerative disease, Diabetes mellitus, Hypertension, Major depressive disorder)

**Fig. 2:**
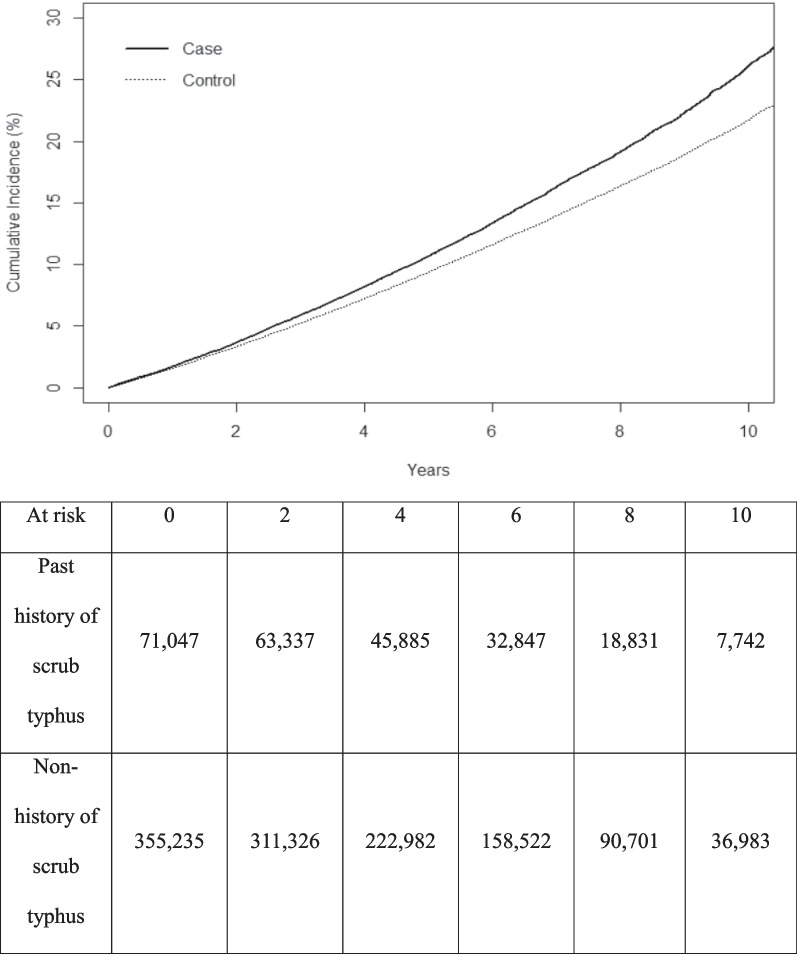
Cumulative incidence of dementia during the follow-up period according to the past history of scrub typhus

The Kaplan–Meier curves for the cumulative incidence of dementia are shown in Fig. 2. It shows dementia incidence in both groups increased over time, and the incidence of dementia was higher in the scrub typhus group than in the non-history of scrub typhus group. As time goes, the gap in the incidence rate also increased.

## Discussion

This nationwide retrospective population-based cohort study found that the history of scrub typhus is related to an increased risk of dementia, especially AD. The prevalence of all comorbidities except hypertension was significantly higher in the scrub typhus group than in the control group. However, the association between a high prevalence of dementia and a history of scrub typhus persisted after the adjustment for age, sex, and comorbidities. This trend became more pronounced over time, that can be attributed to the continued effects of the infection in scrub typhus over time.

Dementia, a degenerative brain disorder, is a major cause of disability. AD is the most common subtype of dementia in the world. Aging is considered an important risk factor as it increases chronic low-grade inflammation, which may be involved in the pathological progress of AD [19–21]. One of neuropathological features of AD is that extracellular neuronal plate containing amyloid beta (Aβ) and neural fiber tangles consisting of intracellular hyperphosphorylation tau filaments, which can be accompanied by deposition of vascular Aβ and inflammation of vessel [22–24]. In AD, several mechanisms contribute to the pathogenesis of neurodegeneration. Among them, neuroinflammation plays an important role in pathogenesis due to mechanisms that exacerbate Aβ and tau pathologies [25–27]. Increased levels of pro-inflammatory cytokines in postmortem brain and the serum of AD patients suggest that neuroinflammation plays an important role in AD pathogenesis [28].

Many studies have suggested that infectious diseases such as bacteria and viruses may be associated with AD development by this neuroinflammatory pathogenesis [29]. This eventually leads to accumulation of Aβ and hyperphosphorylation of tau by inducing excessive neuroinflammation. For example, HSV-1 is the most common cause of viral encephalitis and previous studies have shown that HSV-1 was detected in 70–100% of AD patients over the age of 65. When comparing AD patients with controls, more HSV-1 DNA was identified in AD, which was associated with Aβ plaque [30–33]. This suggests that the recent infection or reactivation of HSV-1 is associated with the pathogenesis of AD. As another example, spirochetes are the bacteria that can cause chronic inflammation in CNS, among which *Borrelia burgdorperi* and *Treponema pallidum* are widely studied [34, 35]. *B. burgdorferi* antigens were observed in neuron and Aβ plaques and neurofibrillary tangles in patients with both AD and neuroborreliosis [34, 36]. According to the study, spirochetal-specific antigens and *T. pallidum* DNA were detected on Aβ plaques, and the risk of AD increased in patients with *T. pallidum* infection [37, 38]. Rickettsial infections can affect CNS, and the most common neurological manifestations are meningitis, encephalitis, and acute disseminated encephalomyelitis [6]. In some studies, rickettsia lasted for a long period in the CNS of immunodeficiency mice and reappeared, causing severe neuroinflammation [39, 40]. This shows that Rickets disease is a potential risk factor for AD. The most representative involvement of CNS in rickettsia is Rocky Mountain spotted fever and epidemic typhus, followed by scrub typhus [41]. Pathological studies of brain autopsy specimens in patients with fatal scrub typhus showed the presence of mononuclear cell infiltration of the leptomeninges and typhoid nodules, microglial clusters, and hemorrhage [42]. These pathological findings are related to the significant upregulation of the pro-inflammatory cytokine gene in scrub typhus infection in murine models. Therefore, this neurotrophic *O. tsutsugamushi* seems to be able to induce neuroinflammation by directly inducing AD through CNS infection, or indirectly inducing AD through various systemic inflammatory effects in the brain.

Although scrub typhus is a disease that causes systemic vasculitis, the relationship between its infection and an increased incidence of vascular dementia remains unclear. Vascular dementia can only be diagnosed if there is sufficient evidence for cerebrovascular disease (CVD) on brain imaging, such as multiple infarctions, subcortical ischemic changes, or hemorrhage [43]. However, systemic vasculitis caused by scrub typhus is usually microscopic and pathologic findings and might not progress to ischemic change to brain parenchyma. Our findings are supported by a previous study that Scrub typhus showed no difference in CVD incidence rate compared to the control group, even though it showed a greater incidence rate of cardiovascular disease when analyzing the National Health Insurance Service-National Sample Cohort data in South Korea [44].

To our knowledge, this is the first to show the long-term progression of dementia-related to a history of scrub typhus infection. Its main strength is its use of medical codes that can minimize interference, such as recall bias and selection bias, and the results could be derived on a large scale. It was also designed to reduce bias through washout time and correct the risk factors for dementia. Because only computerized anonymous information was used, it may be free of ethical problems and personal information leakage.

This study has several limitations. First, since each person’s inclusion or exclusion criteria and diagnosis were made indirectly, such as via operational definitions by code, it is possible that the data may have been over-or underestimated. Second, although some potential risk factors were adjusted for, important risk factors, such as socioeconomic status and family history, could not be considered but may affect the results. Third, after January 20, 2020, there is a possibility of a potential bias due to changes in medical behavior related to the COVID-19 epidemic in Korea.

## Conclusion

In conclusion, our findings suggest that a history of scrub typhus infection in old age is significantly associated with increased dementia risk, especially AD. Additionally, prevention and appropriate treatment of scrub typhus should be emphasized as a preventive measure for dementia.

## Data Availability

All data used in analysis of this manuscript is freely available by contacting the corresponding author.
